# Erlotinib and gefitinib treatments of the lung cancer in an elderly patient result in gastrointestinal bleeding

**DOI:** 10.12669/pjms.295.3661

**Published:** 2013

**Authors:** Hongmei Bai, Qing Liu, Maowei Shi, Jing Zhang

**Affiliations:** 1 Hongmei Bai, First Section of Cadre Ward, General Hospital of the Jinan Military Area Command, Jinan, 250031, P. R. China.; 2 Qing Liu, First Section of Cadre Ward, General Hospital of the Jinan Military Area Command, Jinan, 250031, P. R. China.; 3 Maowei Shi, First Section of Cadre Ward, General Hospital of the Jinan Military Area Command, Jinan, 250031, P. R. China.; 4 Jing Zhang, First Section of Cadre Ward, General Hospital of the Jinan Military Area Command, Jinan, 250031, P. R. China.

**Keywords:** Erlotinib, Gefitinib, Lung cancer, Gastrointestinal bleeding

## Abstract

Lung cancer is currently one of the leading causes of the cancer-related deaths in the world. Erlotinib and Gefitinib are inhibitors of human epidermal growth factor receptor-1 and the epidermal growth factor receptor tyrosine kinase. The most common adverse events for erlotinib and gefitinib were mild to moderate skin toxicity (rash, itching, and dry skin), gastrointestinal reactions (diarrhea and nausea), and fatigue. Erlotinib induced gastrointestinal bleeding is rare, and dose-related. We are reporting a lung cancer patient who received erlotinib and gefitinib. The patient was sensitive to drug and tumor growth was inhibited. However, adverse reactions appeared as drug treatment continued, including gastrointestinal bleeding.

## INTRODUCTION

Lung cancer is currently one of the leading causes of the cancer-related deaths in the world. About 75%-80% of lung cancers are non-small cell lung cancers.^1^^-^^3^ Erlotinib and Gefitinib is an inhibitor of human epidermal growth factor receptor-1 (HER-1) and (or) epidermal growth factor receptor tyrosine kinase (EGFR-TK). As a new aquinas oxazoline compound of low molecular weight, Erlotinib is an oral EGFR-TK inhibitor. Through the inhibition of EGFR-TK autophosphorylation, signal transduction is inhibited to down-regulate cancer cell proliferation.^4^^,^^5^ Erlotinib induces expression of cell cycle inhibition protein p27 to arrest cancer cell cycle in G phase, possibly useful for patients with locally advanced-stage or metastasis non-small cell lung cancers.

## CASE REPORT

A male patient (92-year old) was enrolled in this study. Physical examination showed normal physical development. Superficial lymph nodes were not palpated. Breath sounds were low in both lungs and heart rate was 66 beats/min with regular sinus rhythm. Abdomen was soft with no palpable liver and spleen under rib cage. Shifting dullness was negative. Blood examination showed RBC of 4.32 × 10^12 ^l^-1^, Hb of 102 g/l, WBC of 5.66 × 10^9 ^l^-1^, and N of 67.5%. Chest CT examination showed round nodule in dorsal segment of right lower lung, with largest cross-section of 2.5 cm × 3.0 cm. Multiple sputum cytology suggested adenocarcinoma with blood carcinoembryonic antigen level of 21.88 ng/ml. Results of abdominal B ultrasound was normal. Lung cancer was diagnosed. The patient had no gastritis or acid peptic disease before.

The patient began to take oral erlotinib of 75 mg/d. The original dose was orally taken as 75 mg/day and 112.5 mg/every two days. Cough with hemoptysis sputum was disappeared one month later. On November 5, in chest CT, nodule in right lower dorsal lobe shrunk with maximum cross-sectional as 1.0 cm x 1.5 cm. Blood carcinoembryonic antigen level was 10.06 ng/ml.

Three months later, unbearable itching miliary eruption presented in front chest and limbs, thus erlotinib was reduced to 50 mg/day and 75 mg/every two days. Itching was reduced then. However, vomiting presented and fecal occult blood test was positive. Treatment with erlotinib was replaced for treatment of hemostasis, gastric acid relieving, blood transfusion, and rehydration. Bleeding was stopped on December 16 with negative fecal occult blood test and normal abdominal B ultrasound results.

Symptoms such as vomiting were present. Thus erlotinib was not used any more. A nodule (2.7 cm x 2.0 cm) was detected in the back of the low lobe of the right lung by PET-CT. Therefore, 50 mg/d or 75mg/d of erlotinib was intermittently added in the early September of 2008. On February 15, 2010, pleural puncture was performed and drainage tube was placed in the 7^th^ intercostal space on the posterior axillary line. Regular pleural effusion was extracted, which was bloody.

On February 28, Gefitinib of 125 mg/d was taken orally, and cough and pleural effusion were decreased. On April 9, B ultrasound showed small amount of pleural effusion in right side with maximum anteroposterior diameter of 54 mm, and without abnormal in the left. On July 21, vomiting of 300 ml and positive fecal occult blood test were shown. On July 23, fecal and gastric fluid occult blood test results were all negative. Afterwards, Gefitinib was added, and there were weak positive or positive intermittent fecal occult blood tests. When the drug was discontinued, tests were negative.

## DISCUSSION

The most common adverse events for erlotinib were mild to moderate skin toxicity (rash, itching, and dry skin), gastrointestinal reactions (diarrhea and nausea), and fatigue. Erlotinib induced gastrointestinal bleeding is rare, and dose-related. In this case, the elderly patient was too weak for surgery, chemotherapy or radiotherapy. Thus first-line treatment of single erlotinib was used. The patient was sensitive to drug. Tumor growth was inhibited with initial dose of 75 mg/d to 112.5 mg/d, and lung CT showed tumor shrunk and blood carcinoembryonic antigen level decreased. However, adverse reactions appeared as drug treatment continued, and dose was reduced and then withdran because of intolerance and repeated gastrointestinal bleeding. Since our patient was elderly, the dosages used in this study were lower than those in another report. ^6^ However, our treatment effectively reduced tumor growth, with some adverse reactions such as gastrointestinal bleeding detected. 

After discontinuation of erlotinib, the illness condition became worse and the condition of patient deteriorated, with cough and chest tightness aggravated. Lung CT scan showed large pleural effusion in the right. Another kind of EGFR-TKI gefitinib was taken. It competes with the catalytic domain of MG-ATP to inhibit tumor growth, metastasis and angiogenesis, thus inducing tumor cells apoptosis for anti-tumor.^7^ After treatment with Gefitinib, symptoms relieved rapidly, but drug was intermittently discontinued because of recurrent gastrointestinal bleeding.

The mechanism of erlotinib and gefitinib induced gastrointestinal bleeding is unclear and further study is needed. In this case, dose of erlotinib was increased to 75 mg/ d because of these adverse reactions. Temporary withdrawal or reduction to 50 mg/d is more secure to maintain the disease thus ensure quality of life of the patient. Gefitinib controlled the disease condition, thus clinical symptoms were relieved and survival time prolonged.^8^^-^^11^
